# Strongyloidiasis: An Important Coinfection in the COVID-19 Era

**DOI:** 10.4269/ajtmh.21-0677

**Published:** 2021-09-22

**Authors:** Laura Núñez-Gómez, Belén Comeche, Mercedes Subirats

**Affiliations:** ^1^ Hospital Universitario Ramón y Cajal, Madrid, Spain;; ^2^ Isabel Zendal Emergency Hospital, Madrid, Spain;; ^3^ Department of Microbiology, Hospital Universitario La Paz, Madrid, Spain

A 45-year-old man from Ecuador living for the past two decades in Spain was admitted with coronavirus disease 2019 (COVID-19)-associated acute hypoxic respiratory failure requiring low-flow oxygen therapy and started on dexamethasone 6 mg once daily. Past medical history was unremarkable except for several episodes of suspected allergic reactions with rash and angioedema. The last episode occurred in 2018, and the trigger remained undetermined.

Screening *Strongyloides* serology was requested upon admission and was found to be positive on day 7. The patient’s respiratory condition had improved, and he was otherwise asymptomatic.

**Figure 1.**
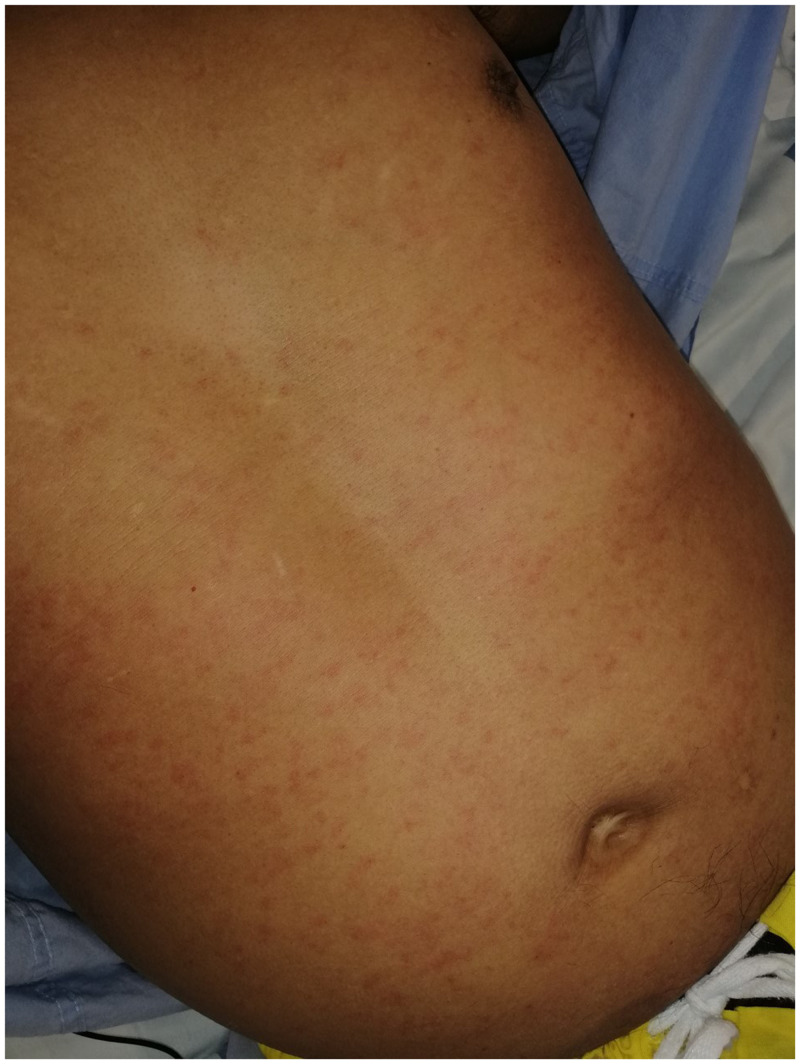
Diffuse maculopapular rash on day 12 of admission. This figure appears in color at www.ajtmh.org.

**Figure 2.**
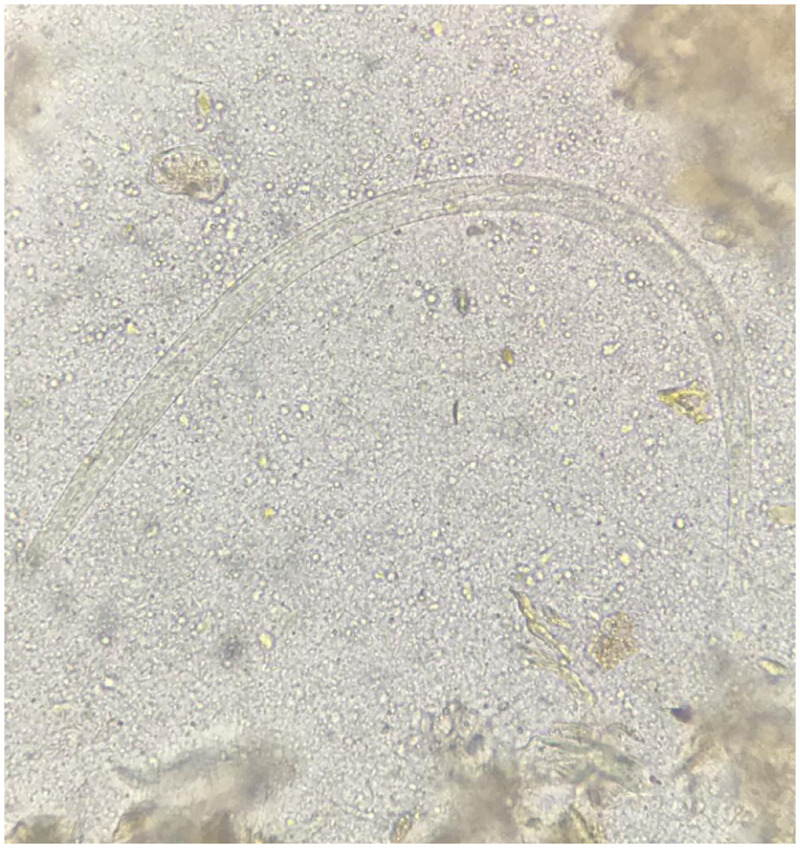
Third-stage filariform larva of *Strongyloides stercoralis* measuring approximately 450 µm. The tail is notched and the esophagus to intestine ratio is 1:1, and hookworm filariform larvae have a pointed tail and a short esophagus. This figure appears in color at www.ajtmh.org.

Stool agar plate culture was requested, and the patient received a single dose of ivermectin 200 µg/kg on day 8 of admission. A pruritic diffuse maculopapular rash appeared on his trunk within hours of administration of ivermectin. An allergic reaction was suspected, and thus corticosteroids were maintained with initial improvement. However, pruritus persisted, and the rash recurred on day 12 (Figure 1). The same day, the result of stool culture was obtained, which was notable for the presence of larvae of *Strongyloides stercoralis* (Figure 2). His respiratory condition was still improving, and he denied gastrointestinal symptoms.

The patient was then diagnosed with *Strongyloides stercoralis* hyperinfection syndrome in the context of corticosteroid therapy. Blood cultures obtained on day 12 to rule out Gram-negative bacteremia were negative. Corticosteroids were stopped after 12 days of treatment, and ivermectin was prescribed. Symptoms resolved within 48 hours of treatment and ivermectin was continued for a total of 14 days.

**Figure 3.**
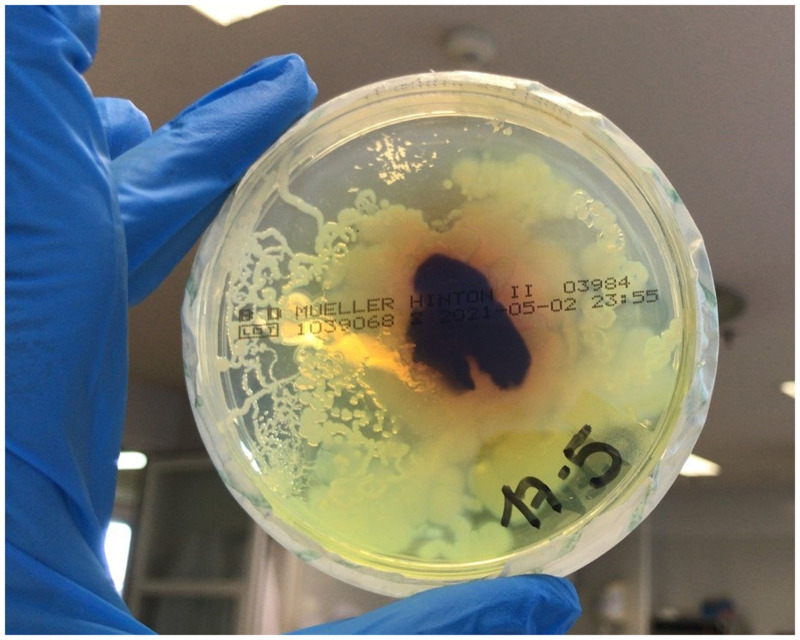
Stool Mueller-Hinton agar plate culture showing growth of bacterial colonies along the larval tracks. This figure appears in color at www.ajtmh.org.

Diagnosis of strongyloidiasis is based on serology and stool testing.^1^ In stool agar plate culture, larvae in stool crawl on the agar, spreading bacteria along their tracks, which then grow in colonies creating patterns (Figure 3).

Strongyloidiasis can be devastating in immunocompromised patients,^2^ and prompt treatment with ivermectin is safe and effective.^3^ Screening is recommended in candidates for immunosuppression – before transplantation or immunosuppressive therapy including corticosteroid treatment – at risk of infection, irrespective of the time spent outside the endemic area.^6^^,^^7^ This becomes particularly relevant with current widespread use of dexamethasone for the treatment of COVID-19.^8^
